# Dentistry among the Ancients

**Published:** 1859-01

**Authors:** 


### ARTICLE IX.

### *Dentistry among the Ancients.*

MESSRS. EDITORS:

As a matter of curious rather than valuable information, I send you the following summary of the knowledge possessed by the ancients with regard to the treatment of the diseases of the teeth and mouth, which you are at liberty to publish if you see proper. It is taken from a translation by Francis Adams, of the works of *Paulus Ægineta,* a celebrated physician, and writer of the seventh century. The work, as is known to medical men, is for the most part, a compilation from the most celebrated ancient writers on medicine. The translation and accompanying commentary, constitutes one of the publications of the Sydenham Society. It will be seen from this, that dentistry has progressed during the last eleven hundred years, as an art, at least quite as rapidly as any of the other specialties of medicine. Indeed, dentistry has won the position it at present occupies as a branch of the curative art within the last half century. From the time of Paulus Ægineta to the commencement of the eighteenth century, it cannot be said to have made scarcely any progress at all. Rude mechanical contrivances have been used as substitutes for the natural teeth, almost from time immemorial. But meohanical dentistry properly speaking, owes the perfection to which it has attained, to the ingenuity of dentists of the last thirty or forty years, as does also the operation of filling teeth, and that there ever should have been a time since the commencement of the practice even of general medicine by intelligent men, when so much ignorance existed with regard to the treatment of the diseases of the mouth as is exhibited in the following extract, is really wonderful.

With the publication of the first edition of Fauchard's celebrated treatise on the diseases of the teeth, "Le Chirurgien Dentiste," in 1728, may be said to have commenced the French school of dental surgery, but in a practical point of view, dentistry made but little progress in this country during the eighteenth century. In England, it may be said to have commenced with the publication of the works of Berdmore and John Hunter, but even here, practical dentistry made but little advancement until after the commencement of the present century. Since then it has been astonishingly rapid. But there is no country in the world where it passed so quickly from incipiency to what it now is, as in the United States. And its present perfection is due as much to the labors of American dentists as to those of any other country. Very respectfully,

D. D. S.

[NOTE.—Although the work from which the following extract is taken, is in the possession of one of the editors, we nevertheless take pleasure in complying with the request of our correspondent, as it will, no doubt, be gratifying to some of our readers who may not have access to the writings of Paulus Ægineta, or the authors from whom his treatises are principally compiled, to know how the diseases of the mouth were treated by the ancients. The prosthesis of the teeth, though sometimes practiced, was certainly but little understood by the ancients, and they were equally ignorant of the eteology and pathology of caries of these organs, as well as of its treatment. The directions given by Celsus and some of the other early writers, for the treatment of toothache, and the diseases of the tonsils and uvula, are very good. But dentistry was practiced to some extent, at a much earlier period even than the time of Celsus. We learn from Herodotus, that the treatment of the diseases of the teeth constituted a specialty of medicine among the ancient Egyptians. Filling teeth with gold must have been practiced by the dentists of Egypt at a very remote period, as would appear from the mouths of some mummies recently found at Thebes. There are specimens of artificial teeth manufactured by them, in the museums of Paris and Berlin, and it is stated that Joseph Mayer, Esq., F. R. S. of Liverpool, has two, one carved in bone, and another of two teeth in wood, fixed in gold. It is also said that Dr. Purland, we believe, of London, has the crown of a tooth found pivoted to a natural root in the mouth of a mummy. Thus, we have abundant proofs of the antiquity of the art, but it had not attained a very high degree of perfection, judging from the information which we have upon the subject.—Eds.]

*On Affections of the Mouth; and, first of the Teeth.*

The teeth are pained without inflammation of the gums, sometimes from pain attacking the body of the teeth, and sometimes from the nerve which enters them being affected. Wherefore they require the strongest remedies; the greater part of which are prepared from the most acrid vinegar. When the gums are pained from inflammation, the best application is oil of lentisk retained in the mouth in a tepid state.

But see that it is new ; for the older it is so much the worse is it for this purpose. This general rule ought to be observed, to evacuate first whatever humor prevails in the general system.

*For inflammation of the teeth.*—Wash with vinegar in which have been boiled galls, or the root of winter-cherry, or the seed or leaves of henbane, or pennyroyal, or the juice of nightshade, or the root of capers, or the leaves of myrtle, or poley, or the root of the wild cucumber, or the leaves of rue with oxymel, or hartshorn, or the vinegar of squills, or pellitory with hyssop. To the eaten part of the tooth apply storax with opium, or galbanum, or sulphur vivum with lycium ; or, let the patient inhale the steam from the seed of henbane through a small funnel. And the antidote of Philo, if applied round the tooth, removes the pain. When there is a defluxion on the teeth, rinse with a decoction of myrtle, lentisk, and galls, or of Syrian sumach, or of the flowers of the wild pomegranate, or of its rind. Sprinkle also of salts two parts ; of burnt alum extinguished in vinegar and pulverized, one part ; then wash with wine. For bloody gums, sprinkle fine alum, or rinse with aloes in wine, or with the root of bramble boiled in wine, or Syriac sumach. When the gums both bleed and are affected with a rheum, burn pickled tunny in a pot until it be reduced to ashes, with which touch the parts. Loose teeth are fastened by being sprinkled with aloes, or Syriac sumach, or fissile alum, or galls, or the root of bramble, either by themselves or boiled in wine.—*Another:* Pulverize the bark of green nuts, and to the expressed juice add Minnæan myrrh and fissile alum, and mix together, and use, by pouring it into the mouth, and put upon the gums of the pained tooth, which it will cure.—*Another:* Triturate together garlic, pepper, and staveacre ; put into a linen cloth ; make small balls of it, and change frequently ; by which means you will purge the humor in the head, and effect a cure of the teeth.

*For loose teeth, running gums, and for every spreading ulcer in the mouth.*—Of burnt chalcitis, dr. xij ; of calamine, dr. viij ; use in powder with vinegar.

*How to remove the teeth without pain.*—Apply flour with the juice of spurge, and above it an ivy leaf, and leave it for an hour. They will spontaneously break in pieces.

*A dentifrice, also for parulis, or gumboil.*—Of that kind of alum called plinthitis, oz. iv; of sal ammoniac, oz. iv; of myrrh, of costus, of pelitory, of each, dr. iv; of pepper, eighty grains.

*For parulis.*—Of sulphur vivum, of pepper, of fissile alum, equal parts. Parulis is an inflammation in a part of the gums, which, not being resolved, suppurates. Having suppurated, and being divided with a scalpel, it is to be kept separate with a tent. Epulis is a fleshy excrescence from inflammation on the innermost dens molaris, being attended sometimes with fever and pain. It must be repressed; and therefore, we must use the species of verdigris called *xyston*, either by itself, or with an equal part of galls, or burnt sori, or burnt alum, or galls alone, or the flakes of copper triturated with vinegar for a sufficient number of days and dried.

*A dentifrice.*—The burnt roots of birthwort, burnt hartshorn, with some mastich.—*Another:* White salts mixed with honey, and wrapped in the leaves of the fig-tree, and burnt until reduced to ashes.—*Another:* Buccinæ filled with salt and burnt; land snails burnt with honey; unwashed wool burnt with a little salts. With each of these, for the sake of fragrance, let there be mixed the schænanth, or spikenard, Indian leaf (*malabathrum*,) or cyperus, or iris.

*An application which will whiten the teeth, repress swelling of the gums, and produce fragrant breath.*—Of pumice-stone, of roasted salts, of iris, of each, dr. iv; of cyperus, dr. v; of spikenard, dr. j; of pepper, dr. vj; pulverize and use.

*For teeth set on edge.*—Painful feeling in the teeth is relieved by chewing purslain, or by rubbing oil of unripe olives, or by lees of oil boiled in a copper vessel to the consistence of honey, and rubbed in after being long kept.

*For worn teeth.*—For worn teeth, apply, of bay-berries, of fissile alum, of the climbing birthwort, equal parts.

*For corroded gums.*—Of the flowers of roses, dr. viij; of galls, dr. iv ; of myrrh, dr. ij. Erosions and running from the gums are cured by washing with asses' milk, the decoction of olive leaves, or vinegar of squills ; or by the following dry applications : the rust of iron, and flowers of the cultivated pomegranate. For swelling and fungous flesh of the gums, the juice of purslain retained in the mouth, the brine of pickled olives, warm oil of unripe olives, or lentisk oil, or oil of apples, or lees of oil, are applicable ; and the following powders ; the rust of iron or copper, the roots of birthwort, the seed of plantain, diphryges, calcined copperas, pomegranate flowers.

*For fissures of the lips.*—Rub with boiled lees of oil. Or this : Of geese fat, with honey and turpentine, equal parts ; of the flowers of roses, of the sorde of unwashed wool, of rose-oil, a small quantity.

*On the disease called ranula.*—Ranula is an inflammatory swelling which forms below the tongue, particularly in children ; wherefore rub the part with equal parts of misy and scraped verdigris (*xyston*) in powder ; and to the chin apply the plasters called *antherum*, *sphærium*, and the *pary grum* from eggs. But, in adults, divide the veins below the tongue in the first place.—*Another :* (it answers likewise with aphthæ :) Of scraped verdigris, of galls, of chalcitis, equal parts. With must it will form a gargle.—*Another :* Having previously rubbed with the flour of tares and honey, anoint with galls triturated in honey, or with the flowers of roses in like manner, or rinse the mouth with a decoction of olive leaves.

*For inflammation of the tonsils.*—If the tonsils and uvula be inflamed in a fever, the most proper gargles are the decoctions of bran, or of roses, or of dates, or of Sebesten plums, or of dried lentil. When the inflammation is at its acme, or is on the decline, we may mix with them some honey, which we must not do at the commencement, nor during its increase, lest, by its acrid nature, it attract a defluxiou. If it suppurate and burst, the patient must gargle with honied water, or with the decoction of lentil, or of roses, persevering until it is completely resolved ; or we may give him to gargle some of the mixtures for rinsing the mouth. If pestilential ulcers in the tonsils take place, we may use the afore-mentioned remedies, and particularly apply the gargle from mulberries with hot water, or hydromel, having the flowers of roses sprinkied upon it, or costus or sumach, either in powder, or in a decoction ; or a decoction of the dried leaves of horned poppy. It is bitter, and it answers best if you dissolve the juice, as we use it for collyria, in honied water. It may also be used with advantage by blowing in the dry herb, or applying it upon the finger. Care must be taken not to touch it with the hand, or, at least, it should be very gently. The trochisk of Andron is also of service. After the irritation from these things is removed, they should use a gargle of liquorice, or that from Scybelitic wine, and of saffron, and of Chian mastich, and of myrrh, and afterwards of starch and tragacanth. When the ulcer has stopped spreading, they may use a gargle of milk and Samian earth. —*Another, for antiades:* Pound sweet pomegranate along with its peel, and mix six parts of its expressed juice with one of honey, boil to the consistence of honey, and anoint.—*Another:* Of immature galls, oz. ij ; of fissile alum, oz. j ; of burnt sal ammoniac, oz. j ; touch with it in powder.—*Another:* Of galls, dr. viij ; of misy, dr. ij ; of roasted salts, dr. v; use in powder. Antias is a scirrhous swelling of the tonsils.

*On the uvula.*—When the uvula is inflamed, we must use the gargles recommended for inflammation of the tonsils, and those of a moderately astringent nature, such as the juice of pomegranate, applied by means of a small spoon, or the surgical instrument invented for the purpose ; and these things may be applied either by themselves, or with moderately boiled honey, or the liquorice root may be mixed with the pomegranate juice. The juice of the liquorice root enveloped in honey also answers well. The swelling may be repressed by the following substances: blood-stone, the Phrygian stone burnt, agerat, the composition from Phrygian stone used for complaints of the pudenda; also Samian earth, Eretrian, Sinopic reddle, the Lemnian earth, the oil of unripe olives by itself, or with some of these; and the fruit of the Egyptian thorn, and fissile alum. The seed and leaves of roses act more mildly, but still more so gum tragacanth, sarcocolla, and starch, which we must use, if pained when repressed by astringents. When the swelling is of equal thickness throughout, in which case we call the disease columella, we must trust to the gargles prepared from myrrh, saffron, and cyperus, and avoid all pressure, and rather anoint with a feather. The following composition answers well: of Syrian sumach, dr. viij; of saffron, dr. iv; of costus, dr. viij; of rose-seed, dr. iv. It may also be applied to the gums. But a thread of a sea-purple color which has been bound round the neck of a viper, and strangled her, has wonderful effects as an amulet in relieving affections of the tonsils and neck, as Galen testifies.

*For hemorrhages from the mouth.*—Apply the pounded leaves of leeks; or dip a new piece of sponge in raw pitch, burn, pulverize, and use. It is also beneficial to rinse the mouth with a cold decoction of green or dried roses, that of vine tendrils, or of the leaves of lentisk, or of bramble, or of quinces, or of roses, or of grape-stones, or of lentils.

COMMENTARY.—All the authors referred to in the twenty-third section may be consulted.

*On the teeth.*—Galen, in particular, deserves to be consulted on diseases of the teeth, which he has treated of very fully in the fifth book of his work 'De Med. sec. loc.' He very properly combats the opinion, which we sometimes hear maintained, that the teeth themselves are devoid of sensibility. He states that, once having toothache, he felt his tooth not only painful but throbbing. One of the best of our modern writers on the teeth, Eustachius, justly remarks that the teeth have a nerve of considerable magnitude distributed upon them, and are in fact possessed of exquisite sensibility. When the teeth ache, Galen says the strongest medicines are indicated, in order to repel and discuss the exciting cause. Most of these are to be prepared with acrid vinegar. He then gives from Archigenes a long list of of compositions for allaying the pain of toothache, from which most of those mentioned by our author are taken. One of the articles which most frequently occurs in them is alum, a solution of which in the spirit of nitre was lately much cried up as a cure for toothache. Of the great number of substances recommended to be put into the hole of the carious tooth it is difficult to form a judgment, as most of them are now never tried in such cases. Some of them seem plausible applications. One consists of pelitory with myrrh; another of opium and pepper; others contain ginger, poppy-juice, hyoscyamus, galbanum, castor, &c. He approves of hot fomentations, and of the heated flour of barley or linseed applied to the cheek. He speaks favorably of filling the hole in the tooth with hot wax. When part of a tooth projects it is to be filed down with an iron file. For pains of the gums he recommends fomentations with vinegar, in which henbane has been boiled. Of dentifrices he has treated at considerable length, and it is from him that our author takes his list.

Scribonius Largus mentions alum among his remedies; and we may remark, by the way, that this medicine is recommended for toothache by many of the earlier modern authorities. (V. Guido de Cauliaco, vi, 2.)

Celsus delivers very judicious instructions for the treatment of toothache. He circumscribes the use of wine, enjoins restricted diet, and food which does not require mastication: then fomentations of hot water by means of sponges are to be applied to the tooth, and so forth. If the pain is more violent, the belly is to be opened, hot cataplasms applied, and some warm liquid retained in the mouth, and often changed. The liquid may be a decoction of some narcotic, such as poppies, mandragora, and hyoscyamus. He praises hot oil applied by means of a probe wrapped round with wool. He also mentions compositions containing pellitory, alum, bitumen, and mustard. He directs us not to be in haste to extract the tooth.

Aëtius gives a variety of applications for removing teeth without an operation. One of them contains red arsenic; another sori and the juice of spurge. Modern dentists are ignorant of such remedies. His account of the nerve which supplies the teeth with sensibility is accurate, but borrowed from Galen.

Octavius Horatianus, like our author, affirms that the juice of spurge (tithymallus) will make the teeth fall out. For the same purpose he likewise praises the power of pelitory and mugwort. After Hippocrates he approves of the application of small bags containing salts or millet.

The medicinal substances recommended by Marcellus, the Empiric, are the same as those already mentioned. He praises strongly a composition consisting of acrid vinegar, alum, and cedar rosin, boiled to the consistence of honey and applied to the carious tooth. To prevent hollow teeth from falling out he directs us to fill the hole with the gum which grows upon the ivy. The juice of poppies pounded in a woman's milk and applied to a carious tooth is said to remove the pain instantly. As a dentifrice he recommends finely powdered glass with spikenard.

Cælius Aurelianus recommends abstinence, rest, and rinsing the mouth with some astringent decoction containing white poplar, mandragora, poppies, henbane with vinegar, hot oil, milk and honey, and the like. He applies bags containing hot flour. If the pain does not abate, venesection is to be had recourse to, a cupping instrument applied near the affected part, and the belly opened afterwards by a clyster. Sometimes the gums are to be scarified or separated from the teeth by means of a scarificator. Respecting anodyne medicines, he remarks that they diminish sensibility, but do not remove pain. He says, like the other authorities, that the juice of the tithymallus, or spurge, breaks the teeth —"dentes infringit." He disapproves very much of early extraction, and mentions that Herophilus and Heraclides of

Tarentum relate cases of persons who had died in consequence of this operation. He says that in the temple of Apollo, at Delphos, there hung a tooth-extractor of lead, which was meant as a hint not to exert great force in extracting teeth. For bleeding of the gums he recommends alum with honey, and the like.

Serapion, like the Greek authorities, mentions a variety of remedial means for diseases of the teeth. One of his prescriptions consists of burnt alum, with vinegar, salt, and sumach. When the pain is violent he directs us to fill the hole with opium or some other narcotic. Avicenna recommends general bleeding, the application of leeches to the gums, opening the ranal veins, and cupping below the chin. His compositions contain opium, burnt alum extinguished in vinegar, galls with vinegar, and the like. He mentions the juice of tithymallus, and several other substances, as possessing the property of making the extraction easy.

Avenzoar recommends, in particular, bleeding from the ranal veins. Mesue's general treatment is very judicious, but similar to that of our author and the others. He also makes mention of alum and vinegar. He says that some apply the actual cautery to the tooth. Haly directs us to heat two needles red hot, and then, having dipped their extremities in oil, to burn the hole of the tooth with them. He recommends us to fill the hole with a composition consisting of pellitory, sal ammoniac, opium, and wax. Some of his applications contain arsenic. That this article would deaden sensibility, and might dcstroy the vitality of the diseased parts, we can readily suppose, but of course it would require to be used with extreme caution. Certainly not more than two grains should be used, and every precaution ought to be taken to prevent the patient from swallowing his saliva. Haly, like most of the others, makes mention of alum. Alsaharavius recommends general bleeding, cupping, scarifications, and leeches ; then warm vinegar, or some warm anodyne infusion, is to be held in the mouth ; or the part fumigated with the vapor of water in which opium, camphor, or henbane has been boiled. He speaks also of the actual cautery. Rhases recommends bleeding, cupping, alum in vinegar, opium, henbane, &c.

The dentifrices and applications to the gums recommended by the Arabians are similar to those of the Greeks. See in particular Haly Abbas (pract. v, 78;) Rhases (Cont. v;) and Alsaharavius (Pract. viii, 2.) Like our authors, theirs contain such astringents and aromatics as balaustine, sumach, galls, spikenard, wild mint, cinnamon, salt of gem, and the like. The pumice-stone in particular was much used for this purpose; but, as Dr. Hill remarks, it is apt to hurt the enamel.

*On ranula.*—Aëtius, Actuarius, and most of the other authorities recommend similar applications. They consist of astringents and escharotics.

Avicenna calls the ranula an enlargement and induration of the sublingual gland. He approves of nearly the same treatment as our author. He recommends in particular burnt vitriol and hermodactylus with the white of an egg. Alsaharavius recommends, in the first place, applications containing nitre, sal ammoniac, and the like. If these do not succeed, caustics are to be applied, so as to occasion a blackening of the part. If this does not answer, an operation must be performed. Rhases makes mention of an application containing green copper, vitriol, &c.

For an account of the manner in which the veterinary surgeons treated ranula in cattle, see Collumella (vi, 8,) and Vegetius (Mulom. iii, 3.) They recommend the tumor to be opened and stimulants applied to it, such as garlic pounded with salt.

The disease which Hippocrates describes by the name of υπογλωσσις appears to have been somewhat different from the one we have been treating of. The hypoglottis of Hippocrates was an inflammatory swelling of the tongue ending in abscess. When matter forms, he directs us to open the abscess. It is also described by Aretæus.

*For inflammation of the tonsils and of the uvula.*—Since, as

Galen remarks, the same treatment applies to inflammation of the tonsils and uvula, we shall treat of both together.

Aretæus has described inflammation of these parts with great accuracy and minuteness. He has also given a very circumstantial account of the ulcers which occur there. Some, he says, are common, mild, and not dangerous ; others are uncommon, pestilential, and fatal. The latter are described as being covered with a livid or black crust. The ulcer, he says, is apt to spread to the tongue, or it passes down the trachea and proves fatal by occasioning suffocation. The disease, he says, is brought on by cold acrid substances, and sympathy with disease of the stomach or lungs. Children are most subject to it. It is endemial in Syria and Egypt. He gives a striking description of death occasioned by suffocation. With regard to the treatment, when the parts are inflamed, swelled, and threaten suffocation, he advises copious bleeding from the arm, acrid clysters, purgatives, ligatures to the extremities, astringent and emollient applications to the parts, cupping the hind-head or breast, and other such means. Respecting the pestilential ulcers, when attended with inflammation and sense of suffocation, he approves of clysters, venesection, gargles, fomentations, ligatures to the extremities, cupping, and so forth. When the disease is spreading, he directs us to burn the sore with powerful caustics, such as alum with honey, chalcitis, and the like. Sometimes, as he remarks, the uvula and parts there are eaten down to the bone.

Galen gives a long and very interesting account of these complaints intermixed with curious extracts from Archigenes. The general treatment consists of venesection, acrid clysters, and purgatives. The local applications are mostly of an acrid and austere nature.

When the tonsils suppurate, Aëtius directs us to open the abscess. He gives an interesting description of pestilential ulcers, which, however, is not very different from that of Aretæus. He approves of bleeding at the arm, suppositories, clysters, ligatures to the extremities, and so forth.

The subsequent Greek authors follow him and Galen in their descriptions.

Marcellus, the Empiric, recommends for swelled uvula various escharotic applications containing chalcitis, flos æris, alum, &c.

Celsus delivers a brief account of ulcers of the internal fauces. He speaks in rather equivocal terms of warm cataplasms, fomentations, and fumigations; but is, upon the whole, inclined to permit the use of them if there be no danger of cold afterwards. He very properly forbids such things as will irritate the parts. He does not approve much of gargles of vinegar, although recommended by Archigenes, whom he calls, "multarum rerum auctor bonus." He prefers emollient gargles at first, and afterwards repellent ones.

The Arabians treat inflammation of these parts like the Greeks. Avicenna follows closely our author's plan of treatment. Mesue approves of bleeding, clysters, and so forth. Rhases mentions bleeding and gargles with vinegar, the water of roses, and other astringents. In inflammation of the uvula, Haly Abbas recommends general bleeding, gentle purgatives, and astringent gargles containing alum, pomegranate flowers, sal ammoniac, and the like; and when these do not succeed, he advises us to have recourse to the operation. (See book sixth.) Alsaharavius recommends a plan of treatment perfectly similar, and, like Haly, directs us to have recourse to excision when other remedies fail. Alexander Aphrodisiensis says, however, that those who have had the uvula cut off die of consumption; and attempts to account for the operation having this effect. (Probl. ii, 3.)

Avicenna and the other Arabians make mention of the amulet recommended by Galen.

[*Paulus Ægineta, sec.* xxvi, *book* iii.

